# Clinical Significance of Cannabinoid Receptors CB1 and CB2 Expression in Human Malignant and Benign Thyroid Lesions

**DOI:** 10.1155/2015/839403

**Published:** 2015-10-11

**Authors:** Eleftheria Lakiotaki, Constantinos Giaginis, Maria Tolia, Paraskevi Alexandrou, Ioanna Delladetsima, Ioanna Giannopoulou, George Kyrgias, Efstratios Patsouris, Stamatios Theocharis

**Affiliations:** ^1^First Department of Pathology, Medical School, University of Athens, Athens, Greece; ^2^Department of Food Science and Nutrition, University of the Aegean, Myrina, Lemnos, Greece; ^3^Department of Radiotherapy, School of Health Sciences, Faculty of Medicine, University of Thessaly, Larissa, Greece

## Abstract

The endocannabinoid system is comprised of cannabinoid receptors (CB1 and CB2), their endogenous ligands (endocannabinoids), and proteins responsible for their metabolism participate in many different functions indispensable to homeostatic regulation in several tissues, exerting also antitumorigenic effects. The present study aimed to evaluate the clinical significance of CB1 and CB2 expression in human benign and malignant thyroid lesions. CB1 and CB2 proteins' expression was assessed immunohistochemically on paraffin-embedded thyroid tissues obtained from 87 patients with benign (*n* = 43) and malignant (*n* = 44) lesions and was statistically analyzed with clinicopathological parameters, follicular cells' proliferative capacity, and risk of recurrence rate estimated according to the American Thyroid Association (ATA) staging system. Enhanced CB1 and CB2 expression was significantly more frequently observed in malignant compared to benign thyroid lesions (*p* = 0.0010 and *p* = 0.0005, resp.). Enhanced CB1 and CB2 expression was also significantly more frequently observed in papillary carcinomas compared to hyperplastic nodules (*p* = 0.0097 and *p* = 0.0110, resp.). In malignant thyroid lesions, elevated CB2 expression was significantly associated with the presence of lymph node metastases (*p* = 0.0301). Enhanced CB2 expression was also more frequently observed in malignant thyroid cases with presence of capsular (*p* = 0.1165), lymphatic (*p* = 0.1989), and vascular invasion (*p* = 0.0555), as well as in those with increased risk of recurrence rate (*p* = 0.1165), at a nonsignificant level though, whereas CB1 expression was not associated with any of the clinicopathological parameters examined. Our data suggest that CB receptors may be involved in malignant thyroid transformation and especially CB2 receptor could serve as useful biomarker and potential therapeutic target in thyroid neoplasia.

## 1. Introduction

Endocannabinoid system (ECS) is an endogenous lipid signal-inducing system, present in various human tissues, that exerts many different and unrelated functions. Substantial studies have indicated the regulatory effects of the ECS on the central and peripheral nervous system, the gastrointestinal tract, and the immune system, being involved in multiple processes, such as gastrointestinal motility, mood, pain regulation, memory, and appetite [1]. These functions are triggered by binding of endogenous and exogenous ligands to cannabinoid receptors (CB receptors). Besides those well-known functions, ECS also exerts antiproliferative effects through modulation of several signaling pathways [2], while its activation may have prognostic significance for tumor developmental progression [3–6].

Two subtypes of CB receptors exist, with different distribution among the human tissues. CB1 receptor is mainly located at the central nervous system, adipocytes, liver, pancreas, skeletal muscle, and T-lymphocytes [7]. CB2 receptor is mainly detected in immune cells, but also in neurons and other cells that comprise the central nervous system such as astrocytes and microglia as well as in cerebromicrovascular endothelial cells [8]. The activation of CB receptors inhibits cAMP formation through its coupling to Gi proteins, resulting in decreased protein kinase A- (PKA-) dependent phosphorylation [7, 8]. CB receptors also couple to extracellular signal-regulated kinase (ERK) and specifically p42/p44 and p38 [2], participating in phosphatidylinositol 3-kinase (PI3K) and ceramide signaling [9]. Other receptors are also attached to the ECS like transient receptor potential cation channel subfamily V member 1 (TRPV-1), peroxisome proliferator-activated receptors (PPARs), and non-CB1/CB2 G-protein-coupled receptors GPR55 [7, 8].

The ECS ligands are the cannabinoids, including the bioactive components of the Cannabis Sativa, synthetic CB-mimetic compounds, and endogenous ligands of CB receptors [10]. The most important molecule of the first category is Δ^9^-THC, which is well known for its psychoactive traits [10]. The other two categories include synthetic ligands that have currently been developed (CP55940, HU-210, HU-211, ab-cannabidiol, ajulemic acid, WIN55,212-2) and endocannabinoids that are produced by the human body and are lipid messenger derivatives of arachidonic acid (AA) conjugated with either ethanolamine or glycerol [11]. The most important of these molecules are anandamide (AEA) and 2-arachidonoylglycerol (2-AG), but additional substances have also been identified, such as O-arachidonoylethanolamine (OAE, virodhamine), 2-arachidonoylglycerol ether (2-AGE, noladin ether), N-arachidonoyldopamine (NADA), and palmitoylethanolamide (PEA) [10, 11]. Apart from the CB receptors and all their ligands, the ECS also includes the essential enzymes for ligand biosynthesis, transport, and degradation [8, 10, 11].

In addition to ECS functions in order to maintain homeostasis, the above-mentioned cross-talk between the ECS and the most important oncogenic pathways (MAPK/ERK and PI3K/Akt pathway) has recently gained interest and has highlighted the significance of the ECS in tumorigenesis [12, 13]. Moreover, cannabinoids have been shown to induce apoptosis in cancer cells, inhibit tumor vascularization via VEGF decrease, and suppress cancer cell invasive capacity [12, 13]. Antiproliferative effects prevail and several studies suggest that cannabinoids have potential as antitumoral agents [12, 13].

Diagnosis of thyroid lesions has recently been increased, not only due to improved diagnostic techniques, but also because of their true incidence rise in the population [14]. In fact, thyroid carcinoma is the most common malignancy of the endocrine system [14]. Although thyroid cancer is usually completely cured by surgery and therapy, 10–20% of patients still die from recurrence or tumor progression [15]. Therefore, it is essential to establish new treatment strategies and find new prognostic markers in order to predict the clinical course for each patient and customize accordingly the available therapeutic modalities. In this aspect, the present study aimed to evaluate the immunohistochemical expression of CB1 and CB2 receptor in benign and malignant thyroid lesions in association with clinicopathological characteristics related to prognosis.

## 2. Patients and Methods

### 2.1. Patients

The examined material consisted of 87 histologically examined thyroid surgical specimens from an equal number of patients who had undergone thyroid surgery for benign and malignant lesions. Forty-three benign (37 hyperplastic nodules and 6 Hashimoto thyroiditis) and forty-four malignant (40 papillary and 4 follicular carcinomas) cases were included in the study. Each neoplasm was classified according to the WHO histological classification of thyroid tumors [16]. The risk of recurrence was estimated according to the American Thyroid Association (ATA) staging system [17]. None of the patients had received any kind of anticancer treatment prior to surgery and there was no clinical history of head and neck irradiation or of other cancer.

### 2.2. Immunohistochemistry

Immunostainings for CB1 and CB2 were performed on formalin-fixed, paraffin-embedded thyroid tissue sections using a goat polyclonal CB1 IgG antibody (N-15, sc-10066, Santa Cruz Biotechnology, Santa Cruz, CA, USA) and a rabbit polyclonal CB2 IgG antibody (H-60, sc-25494, Santa Cruz Biotechnology). Briefly, 4 *μ*m thick tissue sections were dewaxed in xylene and were brought to water through graded alcohols. Antigen retrieval was performed by microwaving slides in 10 mM citrate buffer (pH 6.1) for 15 minutes (min) at high power, according to the manufacturer's instructions. To remove the endogenous peroxidase activity, sections were then treated with freshly prepared 0.3% hydrogen peroxide in methanol in the dark, for 30 min, at room temperature. Nonspecific antibody binding was blocked using Eraser and Sniper, specific blocking reagents for goat and rabbit primary antibodies, respectively (Biocare Medical, Concord, California, USA), for 5 min. The sections were incubated for 1 hour (h), at room temperature, with the primary antibodies against CB1 and CB2 diluted 1 : 300 and 1 : 200, respectively, in phosphate buffered saline (PBS) according to the manufacturer's instructions. Sections were then incubated at room temperature with biotinylated linking reagent (Biocare Medical) for 10 min, followed by incubation with peroxidase-conjugated streptavidin label (Biocare Medical) for 10 min. The resultant immune peroxidase activity was developed using a DAB substrate kit (Vector Laboratories, California, USA) for 10 min. Sections were counterstained with Harris' hematoxylin and mounted in Entellan (Merck, Darmstadt, Germany). Appropriate negative controls were performed by omitting the primary antibody and/or substituting it with an irrelevant antiserum. As positive control, breast and mobile tongue squamous cell carcinoma tissue sections with known CB1 and CB2 expression were used. The follicular cells' proliferative capacity was assessed by Ki-67 immunohistochemical expression, as previously described [18–20].

### 2.3. Evaluation of Immunohistochemistry

Immunohistochemical evaluation was performed by counting at least 1000 tumour cells in each case by two independent observers (Stamatios Theocharis and Paraskevi Alexandrou) blinded to the clinical data, with complete observer agreement. Specimens were considered “positive” for CB1 and CB2 when more than 5% of tumour cells within the section were positively stained [18–20]. The immunoreactivity of the tumor cells for CB1 and CB2 was scored according to the percentage of CB1 and CB2 positive tumor cells as 0: negative staining- 0–4% of tumor cells positive; 1: 5–24% of tumor cells positive; 2: 25–49% of tumor cells positive; 3: 50–100% of tumor cells positive and its intensity as 0: negative staining, 1: mild staining; 2: intermediate staining; 3: intense staining [18–20]. Finally, the expression of CB1 and CB2 was classified as low, if the total score was 0 or 2 and high and if the total score was ≥3 [18–20]. Ki-67 immunoreactivity was classified according to the percentage of positively stained follicular cells exceeded the median percentage value into two categories (below and over mean value), as previously reported [18–20].

### 2.4. Statistical Analysis

Chi-square tests were used to assess the difference of CB1 and CB2 expression between malignant and benign thyroid lesions, as well as between papillary carcinoma cases and hyperplastic nodules. Chi-square tests were applied to assess the associations between CB1 and CB2 expression and clinicopathological characteristics in the subgroup of patients with malignant thyroid lesions. A 2-tailed *p* < 0.05 was considered statistically significant. Statistical analyses were performed using the software package SPSS for Windows (version 13.0; SPSS Inc., Chicago, IL, USA).

## 3. Results

### 3.1. Clinical Significance of CB1 Expression in Human Malignant and Benign Thyroid Lesions

**Figure 1:**
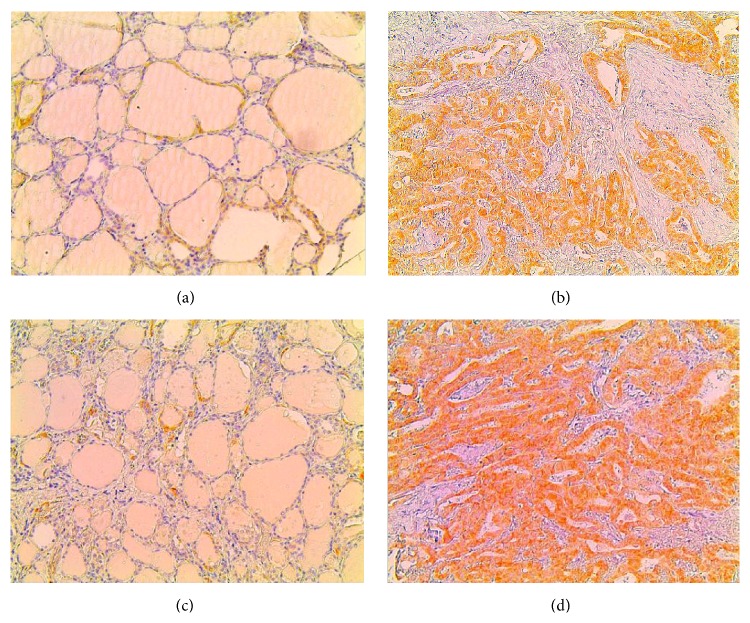
Representative CB1 immunostainings in: (a) hyperplastic nodule and (b) papillary carcinoma. Representative CB2 immunostainings in (c) hyperplastic nodule and (d) papillary carcinoma (original magnification ×200).

CB1 positivity (IHC score > 0) was noted in 52 (60%) out of 87 thyroid lesions. Thirty-one (36%) out of the 87 examined cases presented high CB1 immunoreactivity (IHC score ≥ 3). The subcellular pattern of CB1 distribution was predominantly cytoplasmic and occasionally membranous. Normal surrounding areas adjacent to tumour were found negative for CB1. Representative CB1 immunostainings for hyperplastic nodule and papillary carcinoma are depicted in Figures 1(a) and 1(b), respectively. CB1 immunoreactivity was significantly different between benign and malignant thyroid lesions (Table 1, *p* = 0.0010). High CB1 expression was significantly more frequently observed in papillary carcinoma compared to hyperplastic nodules (Table 1, *p* = 0.0097). CB1 expression was not associated to patients' age and gender and follicular cells' proliferative capacity. In the subgroup of malignant thyroid lesions, high CB1 expression was noted in 23 (52%) out of 44 cases. There was no association between CB1 receptor expression and tumour size, presence of capsular, vascular or lymphatic invasion, lymph node metastasis, and follicular cells' proliferation rate (Table 2). CB1 receptor expression was not associated with risk of recurrence estimated according to ATA staging system (data not shown).

### 3.2. Clinical Significance of CB2 Expression in Human Malignant and Benign Thyroid Lesions

CB2 positivity (IHC score > 0) was noted in 61 (71%) out of 87 thyroid lesions. Thirty-two (37%) out of the 87 examined cases presented high CB2 immunoreactivity (IHC score ≥ 3). The subcellular pattern of distribution was predominantly cytoplasmic and occasionally membranous. Normal surrounding areas adjacent to tumor were found negative for CB2. Representative CB2 immunostainings for hyperplastic nodules and papillary carcinoma are depicted in Figures 1(c) and 1(d), respectively. High CB2 expression was significantly more frequently observed in malignant thyroid lesions compared to benign ones, as well as in papillary carcinoma compared to hyperplastic nodules (Table 1, *p* = 0.0005 and *p* = 0.0110, resp.). In the subgroup of malignant thyroid lesions, high CB2 expression was noted in 24 (55%) out of 44 cases. High CB2 expression was significantly associated with the presence of lymph node metastasis (Table 2, *p* = 0.0301). High CB2 expression was also associated with the presence of capsular, lymphatic invasion, and vascular invasion, at a nonsignificant level though (Table 2, *p* = 0.1165, *p* = 0.1989, and *p* = 0.0555, resp.). No associations between CB2 expression and patients' age and gender, tumour size, and follicular cells' proliferative rate were noted (Table 2). High CB2 receptor expression was more frequently observed in malignant thyroid lesions presenting increased risk of recurrence rate according to ATA staging system, at a nonsignificant level though (*p* = 0.1165).

## 4. Discussion

In the present study, CB1 and CB2 protein expression was increased in malignant compared to benign thyroid lesions. We also describe for the first time an association between CB2 protein expression and clinicopathological parameters crucial for patients' management and prognosis. Notably, enhanced CB2 expression was significantly associated with the presence of lymph node metastases and borderline with the presence of vascular invasion, while indicative but nonsignificant associations with the presence of capsular and lymphatic invasion and estimated recurrence rate were also noted. Similar results for CB1 receptor overexpression were obtained, as far as malignant compared to benign thyroid lesions are concerned; nevertheless nonsignificant association or trend of correlation between CB1 expression and clinicopathological parameters was noted.

In accordance with the present findings, CB receptors were upregulated in certain tumour human malignancies, including oral squamous cell carcinoma, pancreatic, hepatocellular, and prostatic carcinoma, whereas they were not expressed in normal tissues of these organs [3–6]. On the other hand, CB1 receptor was downregulated in colorectal carcinoma in contrast to adjacent normal tissues, pointing to the different roles of the ECS in various tumors and indicating the multiple interactions between the ECS and the mechanisms that control cell growth and proliferation [21]. These mechanisms may include direct induction of transformed-cell death, cell cycle arrest, and inhibition of tumor angiogenesis and metastasis [12, 13]. The antitumoral effects of ECS have also been depicted in numerous studies. In colorectal cancer, endocannabinoids and synthetic cannabinoids were able to induce apoptosis and inhibit carcinogenesis by mechanisms involving both CB receptors, TRPV1 channels and PPAR*γ*-pathway [22, 23]. Similar results have occurred in studies conducted on pancreatic, lung, and breast cancer, cholangiocarcinoma, and hepatocellular carcinoma [24–28]. Synergistic effects of cannabinoids with conventional antitumor chemotherapy have also been reported [29].

CB receptor overexpression in thyroid carcinoma has recently been reported,* in vitro* [30, 31]. More to the point, IL-12 stimulation of anaplastic thyroid carcinoma cell lines induced CB2 receptor overexpression and led to CB2-agonist mediated apoptosis and tumour regression [30]. Moreover, CB2 upregulation rendered the tumour cells more susceptible to treatment with standard chemotherapy [30]. One putative explanation for this phenomenon was the ceramide-dependent activation of the mitochondrial intrinsic pathway, which leads to apoptosis, being triggered by CB2 receptor activation [32]. Another study on thyroid carcinoma cell lines depicted that 2-methyl-2′-F-anandamide (Met-F-AEA) inhibited tumour growth, associated with high CB1 receptor levels [31]. The abundant CB1 receptor expression was noted in more responsive to treatment cell lines, which subsequently were more susceptible to growth inhibition. Such results were ascribed to p53 activation, p21^CIP1/WAF1^ increase, and cyclin A decrease, leading to apoptosis [31].

Apart from the possible therapeutic implications concerning tumorigenesis and ECS, detection of CB receptors overexpression may have potential as prognostic indicators. Upregulation of both CB receptors in hepatocellular carcinoma tissue samples was significantly associated with improved prognosis and longer disease-free survival [3]. Such findings were combined with the histopathological tumour characteristics, as high CB receptor levels were observed in cases presenting well differentiation and limited portal tract involvement [3]. On the other hand, CB2 immunoreactivity was associated with shorter disease-free survival in head and neck squamous cell carcinoma [6]. Concerning CB1 receptor, its overexpression was associated with poor patients' outcome in colorectal, prostatic, and pancreatic carcinoma [4, 5, 21]. Notably, enhanced CB1 expression in stage IV colorectal carcinoma patients was independently correlated with poor prognosis [21]. Increased CB1 expression was also associated with aggressive prostatic adenocarcinoma presenting higher Gleason score, larger tumour size, and increased cell proliferation rate, as well as metastasis at diagnosis [5]. Moreover, low CB1 expression or high FAAH/MAGL levels were correlated with longer survival rate and higher pain status. Similar but not statistically significant results for CB2 receptor were noted [4]. Taking into account the above mentioned data, the currently available studies on CB receptor levels and their associations with prognosis in various tumors seem contradictory and may be closely related to the extent of ECS participation in tumorigenesis.

## 5. Conclusion

Enhanced CB1 and CB2 receptor immunohistochemical expression levels were correlated with thyroid gland malignancy. Moreover, enhanced CB2 expression levels were associated with clinicopathological characteristics important for patients' therapeutic management. These results supported evidence that CB receptors and especially CB2 receptor may interfere with molecular pathways participating in thyroid malignant transformation and could be considered as potential therapeutic targets to suppress tumor progression. Larger cohort studies are strongly recommended in order to confirm and establish the clinical utility of CB receptors as potential prognostic markers in thyroid neoplasia.

## Figures and Tables

**Table 1:** Associations of CB1 and CB2 expression with patients' age and gender, type of histopathology, and Ki-67 protein statement in 87 patients with thyroid lesions.

Clinicopathological characteristics	CB1 expression			CB2 expression		
	Low	High	*p* value	Low	High	*p* value
*N* = 87	56 (64%)	31 (36%)		55 (63%)	32 (37%)	
Age (mean ± SD; yrs)	51.9 ± 14.2	49.5 ± 14.3	0.4584	50.7 ± 14.0	51.7 ± 14.7	0.7620
Gender			0.5470			0.6068
Female	46 (53%)	27 (31%)		47 (54%)	26 (30%)	
Male	10 (11%)	4 (5%)		8 (9%)	6 (7%)	
Histopathology (*N* = 87)			0.0010			0.0005
Benign	35 (40%)	8 (10%)		35 (40%)	8 (9%)	
Malignant	21 (24%)	23 (26%)		20 (23%)	24 (28%)	
Histopathology (*N* = 77)			0.0097			0.0110
Hyperplastic nodules	29 (38%)	8 (10%)		30 (39%)	7 (9%)	
Papillary carcinoma	20 (26%)	20 (26%)		18 (23%)	22 (29%)	
Ki-67 protein statement			0.5051			0.3087
Below mean value	45 (52%)	23 (26%)		47 (54%)	21 (24%)	
Over mean value	11 (12%)	8 (10%)		8 (9%)	11 (13%)	

**Table 2:** Associations of CB1 and CB2 expression with clinicopathological characteristics in 44 patients with malignant thyroid lesions.

Clinicopathological characteristics	CB1 expression			CB2 expression		
	Low	High	*p* value	Low	High	*p* value
*N* = 44	21 (48%)	23 (52%)		20 (45%)	24 (55%)	
Age (mean ± SD; yrs)	51.2 ± 14.4	50.1 ± 14.6		50.2 ± 13.8	51.3 ± 14.9	0.7201
Gender			0.2021			0.9456
Female	15 (34%)	20 (45%)		16 (36%)	19 (43%)	
Male	6 (14%)	3 (7%)		4 (9%)	5 (12%)	
Tumor size (T)			0.6011			0.4844
T1	15 (34%)	18 (41%)		16 (36%)	17 (39%)	
T2–4	6 (14%)	5 (11%)		4 (9%)	7 (16%)	
Lymph node metastasis (N)			0.7132			0.0301
N0	19 (43%)	20 (45%)		20 (45%)	19 (43%)	
N1	2 (5%)	3 (7%)		0 (0%)	5 (12%)	
Capsular invasion			0.8250			0.1165
No	17 (39%)	18 (41%)		18 (41%)	17 (39%)	
Yes	4 (9%)	5 (11%)		2 (4%)	7 (16%)	
Lymphatic invasion			0.5220			0.1989
No	18 (41%)	18 (41%)		18 (41%)	18 (41%)	
Yes	3 (7%)	5 (11%)		2 (4%)	6 (14%)	
Vascular invasion			0.3398			0.0555
No	20 (45%)	20 (45%)		20 (45%)	20 (45%)	
Yes	1 (3%)	3 (7%)		0 (0%)	4 (10%)	
Ki-67 protein statement			0.5827			0.2828
Below mean value	12 (27%)	15 (34%)		14 (32%)	13 (30%)	
Over mean value	9 (21%)	8 (18%)		6 (14%)	11 (25%)	
